# Health and Wellbeing in Aging

**DOI:** 10.3390/ijerph19148835

**Published:** 2022-07-20

**Authors:** Ana Isabel Plácido, Maria Teresa Herdeiro, Fátima Roque

**Affiliations:** 1 Research Unit for Inland Development, Polytechnic of Guarda (UDI-IPG), Av. Dr. Francisco Sá Carneiro 50, 6300-559 Guarda, Portugal; anaplacido@ipg.pt; 2 Department of Medical Sciences, Institute of Biomedicine (iBiMED), University of Aveiro, 3810–193 Aveiro, Portugal

## 1. Introduction

Good health and wellbeing while aging is an ambitious goal proposed by the World Health Organization (WHO) and a core value for most governments [1].

In the last five decades, the development of health technologies very much favored demographic changes. In 2018, the number of people aged 65 and over exceeded the number of children under the age of 5 for the first time [2]. A rise in aging societies is coming, and new efforts are needed to ensure that this increase in life expectancy is accompanied by years of health and good quality of life.

This Special Issue, “Health and Wellbeing in Aging”, aims to compile the most recent studies on interventions that improve older people’s health and wellbeing from different settings and geographical countries to facilitate the dissemination of knowledge in the field and to guide healthcare decision-makers and healthcare providers in developing and implementing strategies that guarantee the promotion of wellbeing with age. This Special Issue covers all areas related to polypharmacy; risk management and the safety of medicines; and the promotion of the health of older adults, such as active aging, e-health, sustainability of aging populations, health service policies, support systems, and pharmaceutical care.

## 2. Aging and Medicines

Age-related comorbidities favor the prescription of multiple medicines, and polypharmacy in older adults easily arises [3]. Polypharmacy is frequently perceived as the overuse of medicines; however, considering that, in some patients, the use of multiple medicines is necessary and beneficial to achieve health outcomes [4], it is useful to analyze the medication used by older adults in terms of clinical appropriateness. The overuse of medicines is associated with the occurrence of drug-related problems (DRPs) namely adverse drug reactions (ADRs). A DRP can be defined as “*an event or circumstance involving drug therapy that actually or potentially interferes with desired health outcomes*” [5]. According to the European Medicine Agency, an ADR is a “response to a medicinal product which is noxious and unintended” [6].

The complexity of pharmacotherapy for older adults increases the challenge of providing appropriate prescriptions to older adults and reinforces the importance of addressing the DRPs in this population. Pharmacokinetic and pharmacodynamic alterations that occur with aging, associated with the use of multiple medicines, predispose older adults to the use of potentially inappropriate medications (PIMs), and ADRs in frail older adults can easily arise [7,8,9]. In older adults, inappropriate prescriptions can be defined as “medications or medication classes that should generally be avoided in persons 65 years or older because they are either ineffective or they pose unnecessarily” [10]. The term inappropriate prescription encompasses potentially inappropriate medications and potential prescribing omissions (POPs) [11].

Considering the high number of medicines used by older adults, polypharmacy, DRPs, and PIMs/POPs have been associated with increases in hospitalization, morbidities, and mortality and with a waste of health resources [12,13,14]. To prevent DRPs and PIM-associated ADRs, several researchers worldwide are developing strategies and using several approaches to promote the sustainability of our aging society.

## 3. Promotion of Wellbeing at Older Ages

Independent of aging health is the cornerstone of life. Because, instead of being associated with better years, longer lives are, in some cases, associated with morbidities, loneliness, and inactivity [15], the World Health Organization has proposed some goals for 2021–2030 related to decreasing health inequities and improving the wellbeing of older adults, families, and communities.

In this context, research groups worldwide are developing new strategies to promote the following:

(a) Healthy aging—according to the WHO—“is a continuous process of optimizing opportunities to maintain and improve physical and mental health, independence, and quality of life throughout the life course [16]. During the ageing process, older adults must be social, economic and culturally active. Active ageing is more than the capacity to perform labour forces” [17].
(b) Health literacy can be defined as a person’s capacity to “gain access to, understand and use information in ways which promote and maintain good health for themselves, their families and their community” [18]. According to the literature, low health literacy is associated with poor health outcomes and higher healthcare costs [19]. In contrast, patients with high health literacy tend to have a more active role in improving their health outcomes and in pushing governments to take actions to address health problems and health and to promote health equity [20,21].
(c) The engagement of older adults with health technology (e-health), specifically the use of e-health tools during everyday life, has been reported in recent studies to be associated with better support regarding their healthcare needs [22,23,24], and in this context, the World Health Organization appealed to the development of strategies to improve digital literacy in older adults [25].
(d) Better decision-making processes and decision-support systems for clinicians can improve the outcomes of hospitalized older adults, according to some studies [26].
(e) Better pharmaceutical care should be provided for older adults through the identification of DRPs and improvements in patients’ literacy [27], with pharmacists having an essential role in patients’ wellbeing due to their proximity to the patients. However, the implementation of pharmaceutical care services can be challenging.

## 4. Final Remarks

Aging societies are one of the massive transformations of the twenty-first century, and health policy measures are needed to ensure that older adults live longer lives in good health. In this search for improvements in the wellbeing of our older population, we must ensure that our health care systems do not burn out.

In this context, the need to develop innovative studies on the above topics is well known, and in this Special Issue, we published these kinds of studies to disseminate scientific knowledge and good practices essential for the healthy aging and wellbeing of older people.
